# COVID-19 and gender inequity in science: Consistent harm over time

**DOI:** 10.1371/journal.pone.0271089

**Published:** 2022-07-08

**Authors:** Mattia Caldarulo, Jared Olsen, Ashlee Frandell, Shaika Islam, Timothy P. Johnson, Mary K. Feeney, Lesley Michalegko, Eric W. Welch

**Affiliations:** 1 Center for Science, Technology and Environmental Policy Studies, School of Public Affairs, Arizona State University, Phoenix, Arizona, United States of America; 2 Department of Public Administration, University of Illinois at Chicago, Chicago, Illinois, United States of America; University of Almería, SPAIN

## Abstract

Stay-at-home-orders, online learning, and work from home policies are some of the responses governments, universities, and other institutions adopted to slow the spread of COVID-19. However, research shows these measures have increased pre-existing gender disparities in the workplace. The working conditions for women during the pandemic worsened due to increased family care responsibilities and unequal distribution of domestic labor. In the academy, working from home has resulted in reduced research time and increased teaching and family care responsibilities, with a larger proportion of that burden falling to women. We investigate the persistence of gender inequity among academic scientists resulting from university COVID-19 responses over time. We draw on two surveys administered in May 2020 and May 2021 to university-based biologists, biochemists, and civil and environmental engineers, to analyze how the pandemic response has disproportionately impacted women in academia and the endurance of those inequities. Results show significantly greater negative impacts from the pandemic on women’s research activities and work-life balance, compared to men. We conclude by discussing the implications of our results, and the need for the academy to better predict and adjust to the gender disparities its policies create.

## Introduction

Women empowerment and gender equality is one of the sustainable development goals global leaders committed to achieve by 2030 [1]. Despite progress in accomplishing this objective, women in the workplace continue to face hurdles. For example, women are shown to still do more unpaid work compared to men, have to cope with gendered organizational environments [2, 3], are usually tasked with a greater share of household responsibilities reducing their work-life balance [4–6]. Women–especially mothers–not only face pressures to be devoted to their jobs, but typically have higher unpaid work costs due to their domestic activities and other family responsibilities [3, 7, 8].

In this unequal context, the COVID-19 pandemic outbreak has left women worse off and increased the amount of unpaid work–such as child and eldercare–they are expected to do [9, 10]. While fathers report increased household chores during the pandemic, mothers increased their childcare and homeschooling responsibilities [11]. One study found that working mothers in the U.K. were 5 percentage points more likely to reduce working hours and 7% more likely to adjust their work patterns than working fathers [9].

While these increased responsibilities fell on women in all careers, gender disparities in academia represent an enduring phenomenon. The scientific and policy debate around its roots and consequences has sharply grown. Inequalities between women and men abound in science, technology, engineering, and mathematics (STEM) academic fields, where institutional and other structural barriers prevent women’s advancements and reinforce gender hierarchies [12, 13]. Although several policies have been enacted to reduce these imbalances, and some progress has been made [13], the COVID-19 pandemic and the measures implemented to slow the spread of the virus may have impaired progress and exacerbated disparities [14, 15].

As we enter the third year of the COVID-19 pandemic, this study investigates the persistence of gender inequity among academic scientists due to formal and informal responses to COVID-19. Data come from two surveys administered in May 2020 and May 2021 to random samples of biologists, biochemists, and civil and environmental engineers employed at Research One (R1) Carnegie classified universities. This study contributes to the gender equality literature by comparing perceptions of how the pandemic affected the paid and unpaid work responsibilities of male and female faculty employed at R1 institutions. Our results from the two-wave survey examine STEM faculty perceptions during and a year after the COVID lockdown, highlighting the enduring effects of COVID-19 policies on gender disparities in science. Results warn policymakers, university administrators, and scientists about the endurance of gender disparities exacerbated by COVID-19 related policies and suggest a fundamental need to develop approaches to crisis response that do not penalize women.

In the next section, we outline the relevant literature on the impacts of gender disparities in academia and how COVID-19 increased them. We then report our data, methods, and findings. We conclude with a discussion of the implications for research and policy.

## Literature review

The COVID-19 pandemic outbreak exacerbated gender disparities, prompting fears of women leaving science [16]. Gender inequality in academia is an international issue that spans scientists’ lifetime, derives from socio-cultural and institutional antecedents, and has harmful consequences for women’s professional and personal lives [17–19]. Literature identifies three key social and environmental factors that harm women’s careers in STEM: cultural socialization processes and stereotypes, chilly academic environments, and socially constructed gender roles [18, 20, 21]. We briefly describe these barriers and how COVID-19 policies have exacerbated them.

Cultural socialization processes and social stereotypes threaten women’s academic careers by repeated exposure to stereotypes depicting women as being less capable in scientific disciplines [21]. These labels reduce women’s interests in science and sustain biases favoring men during hiring, tenure and promotion processes [22]. The lack of female role models in STEM further reinforces these stereotypes, falsely signaling that women are less qualified for succeeding in STEM disciplines [21, 23]. Institutional responses to the pandemic outbreak may have further reinforced these stereotypes and biases. For example, media coverage of COVID-19 has generally enhanced the visibility of male researchers, devaluing women’s contributions and reinforcing the idea that science is unattractive to women [24, 25].

Second, women in STEM often face “chilly” academic environments that are unwelcoming or treat women less fairly than men [26]. As a result, female faculty often do not feel respected or valued and perceive their working environments as discriminatory or sexist [27]. Women are also disadvantaged by informal departmental communication systems which exclude and isolate them [28] in ways that negatively affect collaboration opportunities and undermine professional development and career advancement [29]. COVID-19 may have exacerbated women’s isolation, further reducing the possibilities for network development, professional feedback, or inclusion in new research projects [30].

Socially constructed gender roles are a third element that threatens female scientists’ careers. Women are expected to be more nurturing and caring than men, thus, they are usually assigned greater teaching loads and more advisees [5, 6, 31, 32]. These higher service and teaching loads take time away from research, impairing career advancement. Moreover, women, especially mothers, are expected to take care of domestic activities and to be responsible for the family [20, 33]. At the same time, female faculty experience strong pressures to be exclusively devoted to their jobs [8]. The attempt to satisfy these competing demands arising from these prescriptive images causes stress, often leading to leaving the scientific workforce. The COVID-19 pandemic has further amplified women’s challenges regarding gender roles and the reconciliation of home and work. When the pandemic moved classes online, faculty were forced to adjust syllabi and class materials, but also to integrate work schedules into household routines. The burdens posed by this restructuring compounded by lack of childcare solutions and loss of home assistance fell disproportionately on women, causing additional stress and desire to leave academia [34, 35].

Given the compounding effects of the pandemic on pre-existing conditions penalizing women in science, it comes as no surprise that COVID-19 is leaving female faculty worse off. Since the start of the pandemic, women have slowed research activities, begun fewer new projects, and acquired less research funding—all features essential for academic career advancement [15, 34]. Analyses of published preprints show that between 2019 and 2020 publications authored by men grew faster than those authored by women [14, 36]. Similarly, bibliometric studies report that women have submitted a lower proportion of first author COVID-19 research articles, compared to the proportion of all female first-author articles published before the pandemic [37, 38]. Although the full consequences of the negative effects of COVID-19 policy responses on the research landscape are still unknown, we contribute to the scholarship on gender equity in science and answer the following research question: Did responses to the COVID-19 pandemic cause persistent gender inequities for women in science? Results provide some initial evidence of the endurance of inequities. Moreover, findings have important implications for universities that want to tackle the gender disparities exacerbated by their own policies.

## Materials and methods

### Data collection

SciOPS, a science communication platform developed by Arizona State University’s Center for Science, Technology and Environmental Policy Studies, administered two surveys to tenure-track, tenured, and non-tenure track faculty in biology, civil and environmental engineering, and biochemistry departments at 20 randomly selected R1 Carnegie designated research institutions in May 2020 and again in May 2021. These faculty served as the sample for both surveys. Before administering the second survey, we updated our sample frame, removing faculty who left the university or asked not to be contacted for future surveys. The questionnaire and methodology for this study were approved by the Human Research Ethics committees at Arizona State University (Study #00011868) and at the University of Illinois at Chicago (Protocol #2020–0470). All individuals invited to participate in the study were provided a statement of informed consent. The informed consent language clearly explained respondents’ rights as a research subject. By entering the survey individuals affirmed their consent.

Both surveys were administered in English using Sawtooth Software^®^. The 2020 survey was administered to 1,968 and the 2021 survey to 1,913 researchers, including those individuals who completed the 2020 survey. A total of 362 complete responses were recorded for the 2020 survey and 278 in 2021, resulting in respectively 20.9% and 15.7% response rates as calculated following AAPOR guidelines (RR4) [39]. A total of 143 faculty completing the 2020 survey (39.5%) also responded in 2021. The completed samples were each weighted by gender and academic field to represent the population as closely as possible. A conservative measure of sampling error for questions answered by each full sample is ± 6 percentage points. Student’s t-test, *χ*^2^ tests, and two-way ANOVAs are used to compare survey responses by gender and year. To adjust for multiple comparisons, we only report test findings for p < 0.01. Since attrition represents a form of selection bias which is common in panel and repeated cross-sectional studies [40], we check whether the sample loss between the two surveys may have contributed to attrition bias using two tests described in the framework developed by Fitzgerald and colleagues [41]. Our analyses and estimates are reported in S1 Appendix.

## Lasting effects of COVID on gender disparities in academia

To understand the persistence of gender inequity among researchers working in STEM fields at R1 universities, we first examine descriptive statistics from the two surveys and then look more in detail at the responses provided by scientists who took part in both the 2020 and 2021 surveys. S3–S8 Tables provide the means and standard errors for all the items reported.

### Gender disparities in 2020

Consistent with predictions in the literature [8], and similar to what has been found in other studies [42, 43], results from our 2020 survey indicate that women were more affected than men by increased domestic burdens. For example, compared to men (21.2%), a significantly greater proportion of women (34.3%) reported COVID-19 related policies to have caused unanticipated childcare responsibilities that had a major negative impact on their research (t = 2.7; p < 0.01). The unequal distribution of domestic burdens resulted in more women reporting difficulties concentrating on their work (t = 3.8; p < 0.001), as well as disruptions in preparing publications (t = 2.9; p < 0.01). Tables 1–3 report our estimates: Table 1 shows major negative impacts of COVID-19 on academic life, by gender, Table 2 illustrates gender differences regarding home-life situations, and Table 3 illustrates gender differences with regards to financial problems caused by the pandemic. Taken together, these findings suggest that soon after the pandemic outbreak, women’s personal and professional lives worsened because of the effects caused by the policies put in place to slow the spread of the virus. Although these results are limited to the experience of women working in three specific STEM fields at R1 universities, they support the warnings advanced by some analysts at the beginning of the pandemic on the risk that the rush to safeguard public health may have come at the expense of vulnerable groups and minorities [44].

**Table 1:** Gender differences in 2020 and 2021 regarding the following question: Have social distancing and other COVID-19 related policies had a negative impact on your research in any of the following ways?.

Item	T- Test Mean Difference Estimates 2020	P-Value Mean Diff. 2020	T- Test Mean Difference Estimates 2021	P-Value Mean Diff. 2020
Loss of data	0.075	0.114	0.125	0.016
Loss of biological specimens or animals	0.042	0.202	0.030	0.505
Field work disruptions	-0.014	0.778	-0.019	0.748
Lab work disruptions	0.063	0.196	0.056	0.347
Collaboration disruptions	-0.067	0.212	0.096	0.119
Grant disruptions	0.093	0.074	0.126	0.035
Publishing and other dissemination disruptions	**0.140**	0.004	**0.206**	0.000
Disruptions in student employment	0.001	0.978	0.086	0.157
Disruptions related to administrative or staff employment	0.001	0.978	0.117	0.027
Disruptions due to slow down or university closure	-0.093	0.081	0.126	0.036
Other loss of scientific productivity	0.143	0.027	0.184	0.034

Note: In bold estimates whose p-values are significant (p<0.01).

**Table 2:** Gender differences in 2020 and 2021 regarding the question: Have social distancing and other COVID-19 related policies had a negative impact on your research vis-à-vis any of the following home-life situations?.

Item	T-Test Mean Difference Estimates 2020	P-Value Mean Diff. 2020	T-Test Mean Difference Estimates 2021	P-Value Mean Diff. 2021
Unanticipated childcare responsibilities	**0.133**	0.007	0.141	0.010
Unanticipated elder care responsibilities	0.020	0.395	0.046	0.146
Your own or a family member’s COVID-19 illness	0.005	0.701	0.018	0.447
Anxiety about you or a member of your family contracting COVID-19 disease	0.042	0.359	0.100	0.071
Inability to concentrate on research activities	**0.199**	0.000	0.144	0.014
Other unanticipated complications to homelife	0.030	0.531	0.128	0.077

Note: In bold estimates whose p-values are significant (p<0.01).

**Table 3:** Gender differences in 2020 and 2021 regarding the question: Do you currently have one or more research grants that are facing financial problems that are directly caused by the COVID-19 pandemic?.

Item	T-Test Mean Difference Estimates 2020	P-Value Mean Diff. 2020	T-Test Mean Difference Estimates 2021	P-Value Mean Diff. 2021
Have one or more research grants facing financial problems directly caused by the COVID-19 pandemic	0.042	0.402	**0.164**	0.005

Note: In bold estimates whose p-values are significant (p<0.01).

To check the robustness of our findings, we perform some additional analyses. In detail, for each of the significant estimates reported in Tables 1–3, we estimated logistic regression models controlling for individuals’ field of study (i.e., biochemistry, engineering, biology), institutions (e.g., University of Nevada, Montana State University, etc…), rank (i.e., full professor, associate professor, assistant professor, non-tenured), and whether respondents have been tested for COVID-19. Findings, regarding the 2020 survey are reported in Table 4. Even after controlling for individual and organizational covariates, women are more likely to indicate that COVID-19 related policies have caused publishing disruptions (p<0.01) and inability to concentrate (p<0.01). Estimates for the unanticipated childcare responsibility, however, were not significant anymore at the 0.01 level.

**Table 4:** Logistic model estimating gender differences in 2020 controlling for field of study, rank, and if the respondent tested for COVID-19.

2020 Significant Items	Publishing and other dissemination disruptions		Inability to concentrate on research activities		Unanticipated childcare responsibilities	
	Estimate	P-Value	Estimate	P-Value	Estimate	P-Value
Gender (Reference = Women)	**0.800**	0.009	**0.806**	0.002	0.556	0.050
**Control Variables**						
Field of Study (Reference = Biochemistry)	Yes		Yes		Yes	
Rank (Reference = Assistant)	Yes		Yes		Yes	
University (Reference = Auburn University)	Yes		Yes		Yes	
COVID-19 Test	Yes		Yes		Yes	
N	353		355		356	

In bold estimates whose p-values are significant (p<0.01).

### Gender disparities in 2021

To get a better understanding of the impacts of COVID-19 on gender disparities one year after the initial shutdowns of US universities, in 2021 we asked respondents many of the same questions used in the 2020 survey. We find that in 2021 gender inequalities for women working in biology, biochemistry, and civil and environmental engineering at R1 universities, have remained steady or worsened because of COVID-19. Consistent with bibliometric studies that report women’s reduced productivity during the pandemic [38], we find a greater proportion of women indicated that COVID-19 related policies have caused publishing disruptions (t = 3.6; p < 0.001). Moreover, our results indicate that the pandemic has unequally penalized women from a financial perspective. Compared to 27.0% of male respondents, almost 43.5% of women reported having one or more research grants facing financial problems due to the pandemic (t = 2.8; p<0.01). Tables 1–3 show the 2020 and 2021 data on gender disparities related to academic life, home life, and financial issues.

Also for the 2021 survey, we checked the validity of our estimates by running some logistic regressions and controlling for individual and organizational control variables (i.e., field of study; institution; rank; COVID-19 test). Results are reported in Table 5 and are consistent with the estimates reported in Tables 1–3. In detail, we keep finding that women were more likely to indicate that COVID-19 related policies have caused publishing disruptions (p<0.01) and had them dealing with one or more research grants facing financial problems (p<0.01).

**Table 5:** Logistic model estimating gender differences in 2021 controlling for field of study, rank, and if the respondent tested for COVID-19.

2021 Significant Items	Publishing and other dissemination disruptions		Research grants facing financial problems	
	Estimate	P-Value	Estimate	P-Value
Gender (Reference = Women)	**0.893**	0.003	**0.804**	0.0097
**Control Variables**				
Field of Study (Reference = Biochemistry)	Yes		Yes	
Rank (Reference = Assistant)	Yes		Yes	
University (Reference = Auburn University)	Yes		Yes	
COVID-19 Test	Yes		Yes	
N	277		277	

In bold estimates whose p-values are significant (p<0.01).

### Comparing gender disparities in 2020 and in 2021

To better estimate how gender disparities evolved between 2020 and 2021, we examined several two-way ANOVAs using survey year and respondent gender as factors. To reduce potential biases caused by attrition, we only include data provided by those who responded to both surveys. Results reveal differences by gender but not by year, suggesting long-lasting effects of COVID-19 policies on gender inequalities and supporting what is described earlier in our comparison between 2020 and 2021 survey data.

Compared to men, a greater proportion of women who responded to both surveys reported inability to concentrate on their research activities (F_1,1_ = 16.4; p < 0.0001). Moreover, female scientists were also more likely to report having grant disruptions (F_1,1_ = 8.4; p < 0.01) and research grants that face financial difficulties due to the pandemic (F_1,1_ = 11.9; p < 0.01). Table 6 reports our estimates and shows the extent to which responses to questions on the impacts of COVID-19 policies on the academic life, home life, and financial situation of researchers varied between years and gender.

**Table 6:** Two-way ANOVA regarding the items: Inability to concentrate on research activities; grant disruptions; research grants facing financial problems.

*Items*	*DF*	*F-Value*	*P-Value*
**Inability to concentrate on research activities**			
2021 Survey (1 = yes, 0 = 2020 survey)	1	0.043	0.837
Female (1 = yes, 0 = male)	1	16.428	**0.000**
2021 Survey * Female	1	2.906	0.089
**Grant disruptions**			
2021 Survey	1	1.433	0.232
Female	1	8.372	**0.004**
2021 Survey * Female	1	0.58	0.447
**Research grants facing financial problems**			
2021 Survey	1	0.587	0.444
Female	1	11.851	**0.001**
2021 Survey * Female	1	0.065	0.800

Note: In bold significant p-values (p<0.01).

## Policy implications

Before discussing the insights our results provide into the enduring effects of COVID-19 responses on gender disparities in academia, it is important to acknowledge the limitations of this study. First, our variables measure faculty perceptions of the impact of COVID-19 policies on their research and family lives, lacking more objective measures of these impacts. Future research may address this limitation by looking at how academic publications and grant submissions have evolved during the pandemic and investigating whether gender differences persist over time. Second, while there is some concern about attrition bias across the two surveys, gender differences estimated by looking at 2020 responses are consistent with those obtained from the 2021 survey. This suggests that attrition likely did not seriously affect our results. Last, this study may have limited generalizability as it is focused on researchers working in three STEM fields at R1 universities. However, studies analyzing other researchers (e.g., Matulevicius and colleagues in a 2020 study surveyed medical faculty) find results consistent with ours, suggesting that gender differences caused by the pandemic and its related policies affect the entire academic community and may have long lasting impacts.

Policymakers recognize the negative impacts that the pandemic and COVID-19 related policies have had on gender equality [45]. However, to respond to the emergence of the spreading new COVID-19 variants, governments and universities are enacting policies like those put in place during the first waves of the pandemic—which increased the burdens placed on female academics. This study contributes to the literature on the effects of the pandemic response on gender equality by comparing opinions and perceptions of male and female faculty employed at Carnegie designated R1 institutions. Our results fuel concerns over the enduring effects of COVID-19 policies on gender disparities in science. Our findings suggest that, compared to men, female faculty are more likely to report major negative impacts stemming from the pandemic such as increased domestic burdens, greater financial constraints, and a general reduction of their work-related productivity. Our results call attention to the endurance of these outcomes one year into the pandemic, an important consideration as governments enact policies to confront new COVID variants as we enter year three of the pandemic.

Based on our findings, a greater proportion of women implicate the COVID-19 pandemic outbreak as a cause of grant disruptions and financial problems. To counteract these issues, many federal agencies have been offering no-cost extensions, with some of them also allowing the provision of payments to fellows and trainees unable to work on projects because of COVID-19 [46]. These solutions are helpful to women scientists when funding rules for multiple extensions are flexible and do not have cumbersome paperwork requirements [47]. Additionally, while universities have programs that support faculty in identifying opportunities, preparing applications, and obtaining grant funding, they have not tackled the real problem of work and homelife imbalances that exacerbate inequities for women. Universities should consider devoting more resources to assist faculty with childcare. For example, establishing university daycare centers or facilitating family care at home would provide support for all faculty members—and would remove a significant barrier for female faculty who are more likely to be confronted with unexpected care responsibilities [48]. Providing safe and after-hours access to laboratories (and other academic facilities) would also remove barriers caused by pandemic policies.

Our results show that women report more disruptions to publishing and difficulties in concentrating on their research than men. To reduce these negative impacts, academic departments should ensure women are not overburdened with teaching and service loads [5, 31, 32]. Providing faculty with greater support may allow women to focus more on their research. For example, universities could offer service releases that will not be counted against promotion or performance reviews [47]. In the long run, as policies initially motivated by mitigating the effects of COVID-19 on gender disparities start to wane, universities should consider establishing procedures or mechanisms that continue to aggressively challenge the pre-COVID ‘business as usual’ (e.g., stereotypes, gender roles, biases, chilly academic environments) with the goal of creating a new work culture that eradicates gendered expectations and stereotypes.

Our findings indicate that universities should adapt their policies and procedures to mitigate the impact of future crises on female STEM faculty. Increasing female representation in leadership positions and making faculty and tenure promotion committees more aware of the disparate burdens placed on female faculty are steps that can help universities become more equitable workplaces [49].

Last, our results should inform future considerations regarding some of the lasting changes brought on by COVID-19. For example, universities should pay attention to how the shift towards hybrid work practices will affect gender inequity in science. Will the mix of online and offline work activities increase academic productivity and ease the challenges posed by the need for childcare support, as suggested by some PIs [50]? Will it increase pressure on women to reconcile competing work-life demands, resulting in greater burdens on women’s careers? Answers to these questions deserve greater attention as universities enact new tools and policies to respond to career implications of COVID-19 for women.

## Supporting information

S1 Appendix Attrition analysis. (PDF)

S1 Table Mean differences attritors and non-attritors. (PDF)

S2 Table Attrition probit models. (PDF)

S3 Table 2020 proportion of male and female responding “Major Negative Impact” to the following question: Have social distancing and other COVID-19 related policies had a negative impact on your research in any of the following ways?. (PDF)

S4 Table 2020 proportion of male and female responding “Major Negative Impact” to the following question: Have social distancing and other COVID-19 related policies had a negative impact on your research vis-à-vis any of the following home-life situations?. (PDF)

S5 Table 2020 proportion of male and female indicating they have one or more research grants that are facing financial problems that are directly caused by the COVID-19 pandemic. (PDF)

S6 Table 2021 proportion of male and female responding “Major Negative Impact” to the following question: Have social distancing and other COVID-19 related policies had a negative impact on your research in any of the following ways?. (PDF)

S7 Table 2021 proportion of male and female responding “Major Negative Impact” to the following question: Have social distancing and other COVID-19 related policies had a negative impact on your research vis-à-vis any of the following home-life situations?. (PDF)

S8 Table 2021 proportion of male and female indicating they have one or more research grants that are facing financial problems that are directly caused by the COVID-19 pandemic. (PDF)
