# Supplementary material for: COVID-19 and gender inequity in science: Consistent harm over time
Source: PLoS One. 2022 Jul 8;17(7):e0271089. doi: 10.1371/journal.pone.0271089 (PMC9269954; doi:10.1371/journal.pone.0271089)
Supplement: S1 Appendix — (PDF) [file pone.0271089.s001.pdf]

COVID-19 and gender inequity in science: Consistent harm over time

Supporting Information

S1 Appendix: Attrition Analysis

Attrition represents a form of selection bias which is common in panel and repeated cross-sectional studies [40]. Respondents who decide to opt out from a study may differ significantly from those who continue to participate, introducing bias into the remaining sample. To check whether the sample loss between the two surveys may have contributed to attrition bias we conducted two tests described in the framework developed by Fitzgerald and colleagues [41]. First, we checked whether the characteristics of the scientists who responded to both the 2020 and 2021 surveys were significantly different from those who responded to the first wave but not to the second. We looked at differences in terms of gender, rank, and field. Table S1 shows the results of these analyses. Biochemists ($t = -2.91$; $p < 0.01$) were significantly more likely to drop out and not complete the 2021 questionnaire. Second, we estimated several probit models to identify which of these characteristics were related to the likelihood of not responding to the second wave of the survey when controlling for other variables in the model (see Table S2). The first three columns in Table S2 show the estimated effect of gender, rank, and field alone on the attrition probability, the third column reports the joint estimate of these variables. Our results suggest that compared to biochemists, civil & environmental engineers ($p < 0.01$) were less likely to drop out from the study.
