# Supplementary material for: COVID-19 and gender inequity in science: Consistent harm over time
Source: PLoS One. 2022 Jul 8;17(7):e0271089. doi: 10.1371/journal.pone.0271089 (PMC9269954; doi:10.1371/journal.pone.0271089)
Supplement: S1 Table — (PDF) [file pone.0271089.s002.pdf]

COVID-19 and gender inequity in science: Consistent harm over time

Supporting Information

S1 Table: Mean Differences Attritors and Non-Attritors

Groups	*Retained*	*Opted out*	*T- Test Mean Difference*
**Gender**			
Female	34.3% (47.6)	37.9% (48.6)	-3.6%
**Field**			
Biochemistry	4.2% (20.1)	12.3% (33.0)	**-8.1%***
Civil & Environmental Engineering	26.6% (44.3)	18.3% (38.7)	8.3%
Biology	69.2% (46.3)	69.4% (46.2)	-0.2%
**Rank**			
Full Professor	40.6% (49.3)	39.3% (48.9)	1.3%
Associate Professor	18.9% (39.3)	18.7% (39.1)	0.2%
Assistant Professor	32.9% (47.1)	28.3% (45.2)	4.6%
Non-Tenured	7.7% (26.7)	13.7% (34.5)	-6.0%
N	143	219	

* p<0.01, ** p<0.001, *** p<0.0001

Note: Percentages are presented. Standard errors in parentheses
