# Supplementary material for: COVID-19 and gender inequity in science: Consistent harm over time
Source: PLoS One. 2022 Jul 8;17(7):e0271089. doi: 10.1371/journal.pone.0271089 (PMC9269954; doi:10.1371/journal.pone.0271089)
Supplement: S2 Table — (PDF) [file pone.0271089.s003.pdf]

COVID-19 and gender inequity in science: Consistent harm over time

Supporting Information

S2 Table: Attrition Probit Models

Groups	*Gender*	*Field*	*Rank*	*Overall*
**Gender** (Reference = Male)				
Female	0.098<br>(0.139)			0.125<br>(0.145)
**Field** (Reference = Biochemistry)				
Civil & Environmental Engineering		**-0.876***<br>(0.291)		**-0.920***<br>(0.297)
Biology		-0.641<br>(0.267)		-0.671<br>(0.273)
**Rank** (Reference = Assistant Professor)				
Full Professor			0.073<br>(0.160)	0.018<br>(0.167)
Associate Professor			0.088<br>(0.196)	0.101<br>(0.199)
Non-Tenured			0.445<br>(0.242)	0.441<br>(0.246)
Intercept	**0.231****<br>(0.083)	**0.908***<br>(0.254)	0.173<br>(0.121)	**0.822***<br>(0.293)
N	362	362	362	362
Nagelkerke R^2	0.002	0.036	0.013	0.053

* p<0.01, ** p<0.001, *** p<0.0001

Note: Estimate are presented. Standard errors in parentheses.
