# Supplementary material for: COVID-19 and gender inequity in science: Consistent harm over time
Source: PLoS One. 2022 Jul 8;17(7):e0271089. doi: 10.1371/journal.pone.0271089 (PMC9269954; doi:10.1371/journal.pone.0271089)
Supplement: S3 Table — (PDF) [file pone.0271089.s004.pdf]

COVID-19 and gender inequity in science: Consistent harm over time

Supporting Information

S3 Table: 2020 proportion of male and female responding "Major Negative Impact" to the following question: Have social distancing and other COVID-19 related policies had a negative impact on your research in any of the following ways?

Item	N	Females	Males
Loss of data	356	27.3% (3.9)	19.8% (2.7)
Loss of biological specimens or animals	355	11.3% (2.7)	7.1% (1.6)
Field work disruptions	357	28.4% (4.0)	29.8% (3.1)
Lab work disruptions	360	75.1% (3.9)	68.8% (3.2)
Collaboration disruptions	358	35.5% (4.3)	42.2% (3.3)
Grant disruptions	360	37.5% (4.4)	28.2% (3.0)
Publishing and other dissemination disruptions	359	31.7% (4.2)	17.7% (2.5)
Disruptions in student employment	359	44.6% (4.5)	44.5% (3.3)
Disruptions related to administrative or staff employment	358	19.2% (3.5)	19.1% (2.7)
Disruptions due to slow down or university closure	361	59.3% (4.4)	68.6% (3.1)
Other loss of scientific productivity	225	40.2% (5.4)	25.9% (3.8)

Note: Percentages are presented. Standard errors in parentheses
