# Supplementary material for: COVID-19 and gender inequity in science: Consistent harm over time
Source: PLoS One. 2022 Jul 8;17(7):e0271089. doi: 10.1371/journal.pone.0271089 (PMC9269954; doi:10.1371/journal.pone.0271089)
Supplement: S4 Table — (PDF) [file pone.0271089.s005.pdf]

COVID-19 and gender inequity in science: Consistent harm over time

Supporting Information

S4 Tables: 2020 proportion of male and female responding "Major Negative Impact" to the following question: Have social distancing and other COVID-19 related policies had a negative impact on your research vis-à-vis any of the following home-life situations?

Item	N	Female	Male
Unanticipated childcare responsibilities	362	34.5%<br>(4.2)	21.2%<br>(2.7)
Unanticipated elder care responsibilities	362	5.4%<br>(2.2)	3.4%<br>(1.2)
Your own or a family member's COVID-19 illness	362	1.4%<br>(1.0)	0.9%<br>(0.7)
Anxiety about you or a member of your family contracting COVID-19 disease	362	24.2%<br>(3.9)	20.0%<br>(2.7)
Inability to concentrate on research activities	361	49.0%<br>(4.5)	29.1%<br>(3.0)
Other unanticipated complications to homelife	190	13.2%<br>(4.0)	10.2%<br>(2.8)

Note: Percentages are presented. Standard errors in parentheses
