# Supplementary material for: COVID-19 and gender inequity in science: Consistent harm over time
Source: PLoS One. 2022 Jul 8;17(7):e0271089. doi: 10.1371/journal.pone.0271089 (PMC9269954; doi:10.1371/journal.pone.0271089)
Supplement: S5 Table — (PDF) [file pone.0271089.s006.pdf]

# COVID-19 and gender inequity in science: Consistent harm over time

## Supporting Information

**S5 Table: 2020 proportion of male and female indicating they have one or more research grants that are facing financial problems that are directly caused by the COVID-19 pandemic**

| Item | N | Female | Male |
|---|---|---|---|
| Have one or more research grants facing financial problems directly caused by the COVID-19 pandemic? | 362 | 32.4% (4.2) | 28.2% (3.0) |

Note: Percentages are presented. Standard errors in parentheses
