# Supplementary material for: COVID-19 and gender inequity in science: Consistent harm over time
Source: PLoS One. 2022 Jul 8;17(7):e0271089. doi: 10.1371/journal.pone.0271089 (PMC9269954; doi:10.1371/journal.pone.0271089)
Supplement: S6 Table — (PDF) [file pone.0271089.s007.pdf]

COVID-19 and gender inequity in science: Consistent harm over time

Supporting Information

S6 Table: 2021 proportion of male and female responding "Major Negative Impact" to the following question: Have social distancing and other COVID-19 related policies had a negative impact on your research in any of the following ways?

Item	N	Females	Males
Loss of data	276	28.0%<br>(4.3)	15.6%<br>(2.9)
Loss of biological specimens or animals	276	17.5%<br>(3.7)	14.4%<br>(2.8)
Field work disruptions	272	32.4%<br>(4.5)	34.2%<br>(3.8)
Lab work disruptions	275	67.4%<br>(4.5)	61.9%<br>(3.8)
Collaboration disruptions	277	51.7%<br>(4.8)	42.2%<br>(3.9)
Grant disruptions	277	43.2%<br>(4.7)	30.7%<br>(3.7)
Publishing and other dissemination disruptions	277	45.2%<br>(4.8)	24.5%<br>(3.4)
Disruptions in student employment	278	47.4%<br>(4.8)	38.7%<br>(3.8)
Disruptions related to administrative or staff employment	276	29.2%<br>(4.4)	17.5%<br>(2.9)
Disruptions due to slow down or university closure	278	62.9%<br>(4.6)	50.2%<br>(3.9)
Other loss of scientific productivity	137	46.4%<br>(6.9)	28.0%<br>(4.9)

Note: Percentages are presented. Standard errors in parentheses
