# Supplementary material for: COVID-19 and gender inequity in science: Consistent harm over time
Source: PLoS One. 2022 Jul 8;17(7):e0271089. doi: 10.1371/journal.pone.0271089 (PMC9269954; doi:10.1371/journal.pone.0271089)
Supplement: S7 Table — (PDF) [file pone.0271089.s008.pdf]

COVID-19 and gender inequity in science: Consistent harm over time

Supporting Information

S7 Table: 2021 proportion of male and female responding "Major Negative Impact" to the following question: Have social distancing and other COVID-19 related policies had a negative impact on your research vis-à-vis any of the following home-life situations?

Item	N	Female	Male
Unanticipated childcare responsibilities	277	33.3% (4.5)	19.2% (3.0)
Unanticipated elder care responsibilities	276	9.0% (2.7)	4.4% (1.7)
Your own or a family member's COVID-19 illness	276	4.4% (1.9)	2.7% (1.3)
Anxiety about you or a member of your family contracting COVID-19 disease	275	32.4% (4.5)	22.3% (3.4)
Inability to concentrate on research activities	277	42.0% (4.7)	27.6% (3.5)
Other unanticipated complications to homelife	137	27.0% (6.0)	14.2% (3.8)

Note: Percentages are presented. Standard errors in parentheses
