# Supplementary material for: COVID-19 and gender inequity in science: Consistent harm over time
Source: PLoS One. 2022 Jul 8;17(7):e0271089. doi: 10.1371/journal.pone.0271089 (PMC9269954; doi:10.1371/journal.pone.0271089)
Supplement: S8 Table — (PDF) [file pone.0271089.s009.pdf]

COVID-19 and gender inequity in science: Consistent harm over time

Supporting Information

S8 Table: 2021 proportion of male and female indicating they have one or more research grants that are facing financial problems that are directly caused by the COVID-19 pandemic

Item	N	Female	Male
Have one or more research grants facing financial problems directly caused by the COVID-19 pandemic?	277	27.0% (3.6)	43.48% (4.7)
Note: Percentages are presented. Standard errors in parentheses			
